# Fluid flow induced by helical microswimmers in bulk and near walls

**DOI:** 10.1103/PhysRevResearch.4.033069

**Published:** 2022-07-25

**Authors:** Malay Pal, Itzhak Fouxon, Alexander M. Leshansky, Ambarish Ghosh

**Affiliations:** 1 Centre for Nano Science and Engineering, Indian Institute of Science, Bangalore 560012, India; 2 Department of Chemical Engineering, Technion – Israel Institute of Technology, Haifa, 32000 Israel; 3 Department of Physics, Indian Institute of Science, Bangalore 560012, India

## Abstract

Magnetic nano- and microswimmers provide a powerful platform to study driven colloidal systems in fluidic media and are relevant to futuristic medical technologies requiring precise yet minimally invasive motion control at small scales. Upon the action of a rotating magnetic field, the helical microswimmers rotate and translate, generating flow in the surrounding fluid. In this paper, we study the fluid flow induced by the rotating helices using a combination of experiments, numerical simulations, and theory. The microhelices are actuated either in a fluid bulk or in proximity to the bottom wall using typical microfluidic device setup. We conclude that the mean hydrodynamic flow due to the helix actuation can be closely approximated by a system of rotlets line distributed along the helical axis (i.e., representing the flow due to rotating cylinder) which gets modified close to a wall through appropriate contributions from image multipoles. As the mean flow can be obtained in closed form, this study can be further applied towards modeling of the dynamics in a swarm of driven microswimmers interacting hydrodynamically near a bounding surface.

## I Introduction

Achieving controlled motion in fluids and gels with nano-(micro)scale objects, referred to as nano-(micro)swimmers, can lead to innovative technologies pertaining to biomedicine [1–10], on-chip assembly [11–13], environmental remediation [14,15], and microfluidic manipulation [16–24]. In addition, understanding the collective motion of nano- and microswimmers is of great fundamental interest, since they mimic various intriguing natural phenomena, such as bacterial colonies [25], flock of birds [26], school of fish [27], swarming insects, and even migration of human population. Since motion at small scales is dominated by friction as well as randomization due to thermal fluctuations, specialized techniques are necessary to achieve controlled motion. To date, there have been numerous strategies to develop artificial swimming strategies, relying on chemically [28], acoustically, electrically, optically [29], and magnetically [12,18] driven colloidal particles. Of these, magnetically driven actuation has been particularly attractive due to its noninvasive nature, especially with respect to living systems.

The focus of study in this paper is magnetically driven motion of rigid, nano-(micro)scale ferromagnetic helices. The motion was powered by applying a rotating magnetic field to turn helices that translate due to rotation-translation coupling [18,30–33]. This strategy has been applied to achieve controlled motion in various environments, including those of high biological relevance [34]. Depending on the method of fabrication, these swimmers can also be rendered multifunctional and have demonstrated useful in biophysical measurements [7–9,35], therapeutics [36–38], microfluidic manipulation [39], and other applications. When the rotating magnetic field is replaced by an oscillating drive, the helical microswimmers perform back and forth motion exhibiting enhanced diffusivities [40,41]. Understanding the collective dynamics of this novel artificial active matter system is of fundamental interest, since the dominant interaction between the swimmers is of hydrodynamic origin, similar to many biological self-propelled microorganisms. The activity of the artificial system can be readily controlled using externally applied magnetic fields, which allows easy experimentation.

Previously, the dynamics of helical microswimmers in different fluids, including shear-thinning [16] and viscoelastic media [42,43], has been thoroughly investigated. However, how the microswimmers influence the host medium through inducing a motion in the surrounding fluid has not yet been studied in enough detail [44], in particular how the induced flow is modified due to a boundary. Also important is the representation of the flow with analytical models, both in bulk as well as near a surface. This is necessary for understanding and predicting the motion for helical swimmers in the presence of passive interfaces, obstacles, and colloids dispersed in the suspending medium, as well as understanding the role of hydrodynamic interactions in their collective behavior. Previous studies of similar nature [45] have been focused on helical bacteria which are force- and torque-free objects, as compared to externally powered helical swimmers that are driven by an external magnetic torque and therefore generate different flow in their surroundings.

The paper is organized in the following manner: first, we describe the experimental system and methodologies used for measurements of the induced flow. We then present experimental results of the flow profile in a plane perpendicular to the helical axis and show the measurements to be in good agreement with numerical simulations. The fluid flow was then represented by single pointlike as well as line-distributed rotlets and compared with previous reports. Next, we show experimental and numerical results with helical swimmers moving close and parallel to a surface and present a semi-analytical model to describe the fluid flow accurately.

## II Experimental System And Methodologies

**Fig. 1:**
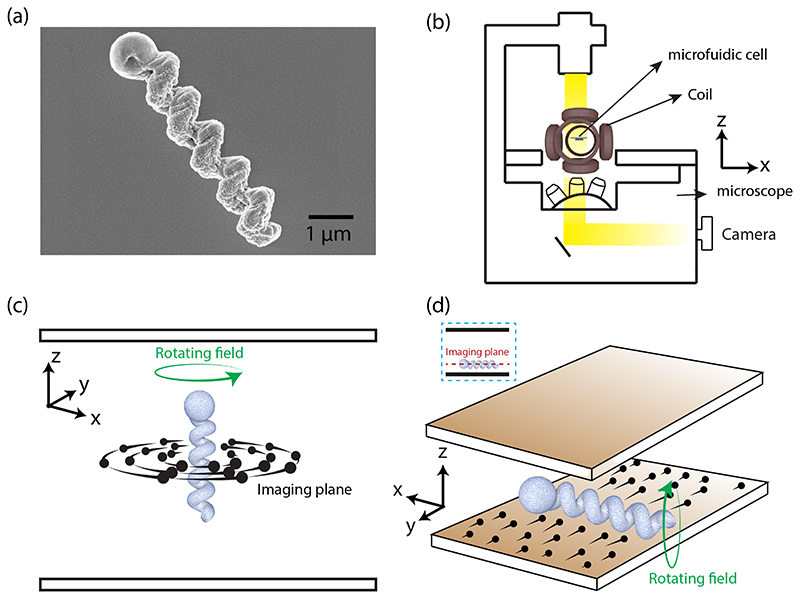
Experimental system and strategy. (a) Scanning electron micrograph of a microswimmer, scale bar 1 *μ*m. (b) Schematic of the experimental setup: an optical microscope, magnetic coil, and the microfluidic device. (c) Experimental strategy that uses tracer particles (black dots) to map the flow induced by a microswimmer in the bulk fluid away from walls. (d) Experimental strategy to map the flow induced by a microswimmer actuated near one of the chamber walls. The tracer particles (black dots) and their trajectories are also depicted; the imaging plane is shown in the inset.

The helical microswimmers used in this experiment were geometrically similar to previous studies [32,33,46]. As shown in scanning electron micrograph in Fig. 1(a), the helices were 5 *μ*m long and had 0.6 *μ*m wide, with axial pitch of 1 *μ*m, and flagellum radius around 0.3 *μ*m. The nanohelices were fabricated using glancing-angle deposition on a monolayer of 1–1.2-*μ*m polystyrene (PS) beads deposited on a silicon substrate. The helically nanostructured thin film was sonicated in deionized water and drop casted on a freshly piranha-cleaned silicon wafer which was left for drying for 24 h. Once the water evaporated, we deposited 50-nm film of magnetic material (here, iron) on the microswimmers. Energy-dispersive spectroscopy image of a microswimmer is shown in Fig. F1 of Supplemental Material [47], confirming the presence of the constituent materials.

A microfluidic chamber of Hele-Shaw geometry containing microswimmers was placed inside the triaxial Helmholtz coil integrated with an optical microscope, as shown schematically in Fig. 1(b). To investigate the fluid flow induced by the swimmer in bulk liquid far from any surface, we use the strategy shown in Fig. 1(c). The plane of the rotating magnetic field was parallel to the chamber surface, such as to propel the swimmer upward. The swimmer could move upward once the thrust due to the rotating field was larger than the downward force due to the weight of the helical structure, which typically occurred at frequency *f_B_* ~ 10 Hz for magnetic field strength of 20 Gauss. The height of the fluidic chamber was around 100 *μ*m, and the plane of imaging was midway, approximately 50 *μ*m above the bottom surface. As the swimmer moved through this location, the fluid flow induced in the plane intersecting the center of the helix was filmed and subsequently analyzed. We also investigated the fluid flow induced by the microswimmers moving close to the bottom surface, as shown schematically in Fig. 1(d). Here, the plane of the rotating field was kept parallel to the chamber surface.

**Fig. 2:**
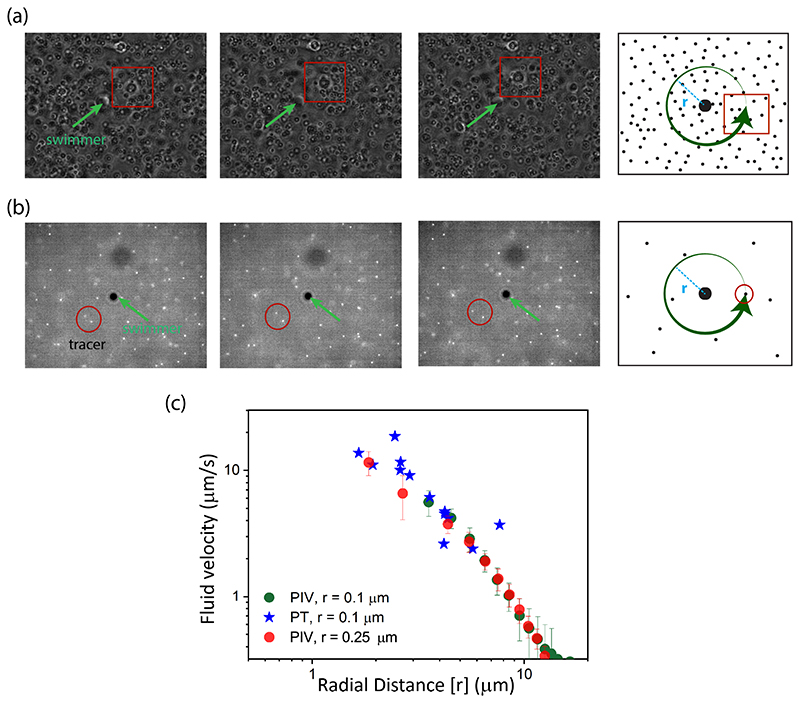
Analysis of the fluid flow. (a) Screenshots of the tracer particles along with the representative block used for PIV analysis, along with the schematic representation of the method used. (b) Particle-tracing technique (PT) to estimate the fluid flow, showing experimental snapshots and schematic of the process. (c) Comparison of the fluid flow estimated by the two methods (PIV and PT) as a function of the radial distance in the imaging plane. The symbols correspond to different techniques and size of tracer particles.

To analyze the fluid flow induced by the microswimmer, we employed and compared two techniques, as described below. In the examples shown here, the swimmer propelled upward within the chamber [as shown schematically in Fig. 1(c)], corresponding to swimming in the bulk. The same techniques were also used for estimating the flow induced by microswimmers propelled close to the bottom surface (shown later). In the first technique, shown in Fig. 2(a), we used particle image velocimetry (PIV) for measuring the fluid flow experimentally using 500- and 200-nm PS beads as tracer particles. To suppress the effect of Brownian motion, we used glycerol-water mixtures of different viscosities *(η* = 30 cP for 0.50-*μ*m beads, 50 cP for for 0.2-*μ*m beads). Images were preprocessed with background subtraction, and then fast Fourier transform window deformation (with multiple passes: 124 × 124 and 64 × 64 with 50% overlap) algorithm of MATLAB open-source library PIVLAB was utilized for the PIV analysis. In the second approach, we measured the fluid velocities by tracking the tracer particles directly from the video frames. In these experiments, the volume fraction of the tracers was kept as low as 10^−4^ [see Fig. 2(b)]. The two tracking methods yielded very similar results, as shown in Fig. 2(c), in the variation of the fluid flow speeds generated by the swimmer as a function of distance. Note that the methods adopted here provide flow profiles in the plane defined by the imaging system. The depth of imaging of the microscopy system using 100×, 1.4 numerical aperture (NA) objective was approximately equal to the diameter of a single bead, allowing us to track a monolayer of beads located at the same height in the chamber. The measurement with fluorescent PS beads of diameter 0.2 *μ*m (green circles) in the same graph [see Fig. 2(c)] confirms their suitability in flow measurements.

**Fig. 3:**
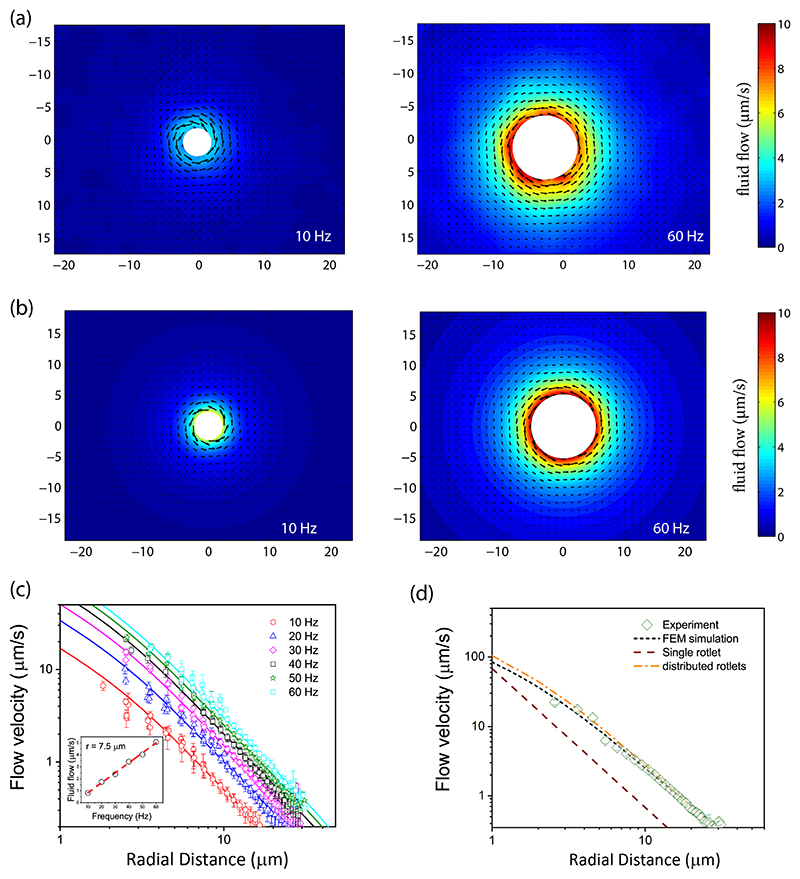
Flow induced in bulk fluid. (a) Experimentally estimated magnitude of fluid-flow velocity for helices rotating about their long axis at frequencies 10 and 60 Hz, in a plane perpendicular to the long axis of the swimmer. The white area close to the helix denotes the region where the tracer particles move very quickly, such that it was not possible to estimate their speed accurately using PIV. (b) Corresponding fluid flows estimated from finite-element simulations under similar conditions as described in main text. (c) Comparison of experimental (symbols) and simulated (solid lines) fluid velocities as a function of the radial distance, ***r***, from the helical axis in the midplane passing through swimmer’s center, at different frequencies. Error bars represent the standard deviation. The inset shows the anticipated linear dependence of the flow magnitude vs frequency at ***r*** = 7.5 *μ*m. (d) Fluid flow induced by swimmer at 50 Hz as a function of the radial distance. Experimental and simulated data are well represented by line-distributed rotlets. Also shown in red dashed line are predictions corresponding to a point rotlet.

For measurements in the fluid bulk, microswimmers were first transported to the midplane of the microfluidic chamber and then filmed as they move either upward or downward. The filming stopped once the swimmer’s long axis went out of the focal plane. The fluid flow in the plane perpendicular to the helix axis was probed and analyzed when the helix was located around the imaging plane. The magnitude of the fluid flow in the planes perpendicular to the long axis was relatively insensitive around the midsection of the helix, which allowed us to time average the flow speeds and thereby improve the signal-to-noise ratio. The averaged experimental flow profiles for microswimmers rotating at *f* = 10 and 60 Hz are shown in Fig. 3(a). The fluid flow was essentially rotational around the helical axis, increased at higher rotation speeds, and reduced at distances far from the helix. Experimentally measured flow profiles were then compared with calculations for the fluid flow using finite-element method (FEM) simulations of the Stokes equation, η∇^2^***u*** = **∇***p*, where ***u*** and *p* are velocity and pressure fields, and *η* is the dynamic viscosity of the incompressible Newtonian liquid. For FEM simulations, we consider 5-*μ*m-long helix with *a* = 0.3 *μ*m helical radius, filament radius 0.3 *μ*m and pitch 1 *μ*m. The use of the Stokes flows equations can be justified by calculating the corresponding Reynolds number, *Re* = Ω*a*^2^*ρ*/*η*, where Ω is the angular frequency (=2π*f*) and *ρ* the fluid density. For the highest frequency *f* = 60 Hz and the less viscous solvent with *η* = 30 cP, we estimate that *Re* ≈ 10^−6^ ≪ 1. In the results shown in Fig. 3(a), the head was not considered in the analysis of the flow, since its contribution was negligible, as shown in Fig. F2 of the Supplemental Material [47]. The simulations were performed in a cubic domain with linear dimensions 200 times the length of the helix, which ensured negligible effect of the boundary on the fluid flow. The mean (averaged per one revolution of the helix) flow solutions were obtained by considering the rotating helix at four different orientations, corresponding to rotation by 0°, 90°, 180°, and 270° about the helical axis, and thereafter averaging over the four orientations. We used COMSOL MULTIPHYSICS package to implement these simulations. The simulated flow profiles for 10- and 60-Hz rotation rates are depicted in Fig. 3(b). In Fig. 3(c), we plot the magnitude of the flow velocity as a function of the radial distance from the swimmer’s long axis at different rotation rates and find excellent agreement between the experimental and simulation results (also see Fig. F3 of the Supplemental Material [47]). As expected from linearity arguments, the flow magnitude was found to be proportional to the rotation speeds (as shown in the inset). From both the experimental and the numerical data, we found that the fluid velocity decays as a power law ~*r^–a^* with a distance-dependent exponent 1 < α < 2 (e.g., *α* ≈ 1.75 for the radial distance *r* = 10 *μ*m). This is reasonable considering that the flow induced by a point rotlet and by an infinitely long cylinder rotating about its axis decays as ~*r*^−2^ and ~*r*^−1^, respectively, so that the exponent in the decay rate of the flow induced by a rotating helix of a finite length would be between these two limiting values.

## III Results And Discussion

Next, we investigate ways to represent the flow analytically. First let us consider a point rotlet [48,49] solution of Stokes equations given by

(1) $$u_{i}=\frac{T_{j}}{8\pi \eta }\frac{\epsilon_{ijk}r_{k}}{r^{3}},$$

where ***r*** is the radius vector from the source and repeated indices imply summation. The rotlet strength *T_j_* can be related [48] to viscosity *η*, angular velocity Ω_*j*_ using $\frac{T_{j}}{8\pi \eta }={\text{Ω}}_{j}a^{3}$, where *a* has the dimensions of length. We show the theoretical prediction of the flow induced by the point rotlet positioned at the middle point of the helical axis for *f* = 50 Hz in Fig. 3(d). We chose *a* = 0.6 *μ*m, which provided a good match between numerical and experimental results. However, for a point rotlet the flow velocity falls off much faster away from the helical axis. The more accurate representation of the flow is given by a line distribution of rotlets along the helical axis (i.e., *z* axis), given by

(2) $$u_{i}=\int_{-l}^{l}M_{j}G_{ij}dz^{\prime },$$

where $G_{ij}=\frac{\epsilon_{ijk}r_{k}}{r^{3}}$, with ***r*** = *(x, y, z–z’*) and the coordinate origin at the helix center at *z* = 0. Considering the system of rotlets to be uniformly distributed between *−l* and *+l* along the *z* axis, then after integrating and applying the boundary conditions at the outer (cylindrical) envelope enclosing the helix, i.e., *u_x_* = –Ω_y_, *u*_y_ = Ω*x* at *y*^2^ + *x*^2^ = *a*^2^, we obtain the fluid velocity (see Supplemental Material [47], Sec. S1):

(3) $$\begin{array}{c} u_{i}=M\epsilon_{i3k}\frac{x_{k}}{\left(x^{2}+y^{2}\right)}\{\frac{l-z}{\sqrt{x^{2}+y^{2}+{\left(l-z\right)}^{2}}}\\ +\frac{l+z}{\sqrt{x^{2}+y^{2}+{\left(l+z\right)}^{2}}}\}, \end{array}$$

with a constant torque density $M=\text{Ω}\frac{a^{2}}{2}$. The flow velocity in Eq. (3) shows an excellent agreement with the experimental and numerical flow profiles for *a* = 0.6 *μ*m [see Fig. 3(d)]. Notice that in Figs. 3(c) and 3(d) the maximal radial distance is about *r* ~ 30 *μ*m, meaning that we cannot observe the anticipated far-field *~r*^–2^ decay of the flow field that should take place at distances *r* ≫ 2*l* ~ 10 *μ*m.

To summarize the experimental and numerical results for helical microswimmers in bulk, rotational flow is induced by a helix twirling about its long axis, which can be analytically described by a system of line-distributed rotlets. For a chiral object such as helix, (as opposed to a cylinder), there will also be a longitudinal flow *(u_z_*) in the direction of the helical axis. As found by the simulations (in Fig. F4 of the Supplemental Material [47]), the magnitude of the longitudinal flow is much smaller than the flow in plane transverse to the long axis in Eq. (3). We will revisit the issue of the longitudinal flow below. Please also notice that our theoretical analysis relies on a flow induced by a rotating cylinder rather than a helix, while there will be differences in the local flow field due to the local helical geometry (see Fig. F5 of the Supplemental Material [47]). This would, however, be only important at proximity to the swimmer’s surface (i.e., at length scales comparable to the helix radius) and therefore expected to have negligible hydrodynamic effects at larger distances.

**Fig. 4:**
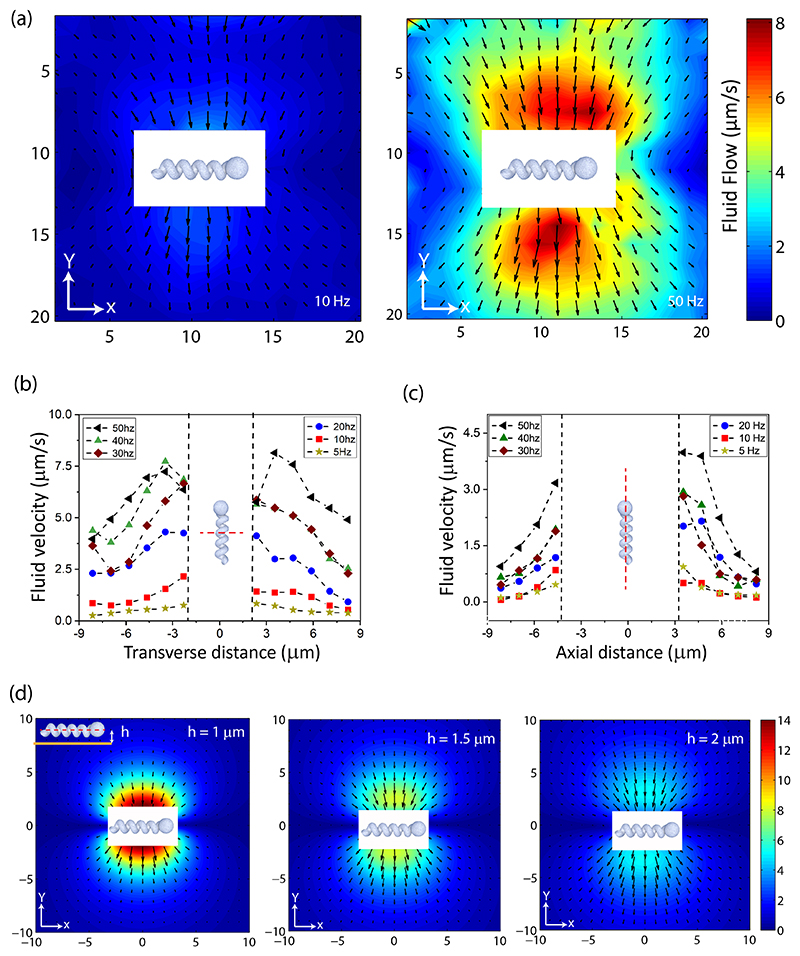
Flow field of a swimmer aligned parallel and located near the bottom of the chamber, moving from left to right. The white box around the swimmer denotes the region where the tracer particles move in and out of focus, which makes it difficult to accurately measure their speed. (a) Experimental flow profiles at 10- and 50-Hz rotation speeds. (b) Flow speed as function of transverse distance (along the red dotted line in the inset image) from the helix for different rotation rates. (c) Same as (b), for flow along the helical axis (along the red dotted line in the inset image). (d) Numerical simulation of the fluid flow at different distances between the microswimmer and bottom surface. We assumed the helix to be rotating at 30 Hz and having propulsion (along the helical axis, left to right) and drift (perpendicular to helical axis) velocities the same as the experiment.

In typical situations, the microswimmers are heavier than the surrounding fluid, implying that they tend to sediment and propel close to a bounding surface. To investigate this important phenomenon experimentally, we left the microfluidic chamber undisturbed for a few minutes after placing it inside the Helmholtz coil, which ensured that all microswimmers settled down to the bottom of the chamber. The chamber made of glass coverslips was treated with acetone and isopropyl alcohol for 3 min each in an ultrasound bath. This keeps the glass surface hydrophobic but cleans the surface of the coverslip as required, with minimum sticking of the microswimmers to the surface. Standard PIV tracking was difficult to implement in this case because the individual microswimmer was steadily moving within the imaging plane (as compared to motion perpendicular to the imaging plane in experiments far from the surface as described before. For example, the propulsion speed of the helix along its axis was about *U_prop_* = 0.8 *μ*m/s, at 10-Hz rotation rate. Due to interaction with the surface, it also exhibited a drift (perpendicular to its long axis) with velocity *U*_drift_ = 0.6 *μ*m/*s*. We therefore performed the PIV analysis in a comoving frame of reference affixed with the microswimmer. This allowed averaging over many frames (>50), which in turn reduced the effect of the Brownian motion of tracer particles. To estimate the fluid flow in the lab frame, shown in Fig. 4(a) for two rotation rates, we compensate the vector flow fields with the constant mean propulsion velocity of the swimmer. In Figs. 4(b) and 4(c), we plot the fluid velocity as a function of the radial and axis distances, respectively, for various rotation rates.

There are several features to be noticed from these plots: (i) there is a slight fore-and-aft asymmetry in the magnitude of the flow; see Figs. 4(a) (right plot) and 4(b). This probably occurred because the bottom surface was not perfectly parallel to the imaging plane. The fluid speeds are quite sensitive to the distance from the surface; thus, a small differential in the direction transverse to the helical axis could produce quite a difference. To confirm this hypothesis, we changed the direction of motion and observed the change in flow asymmetry depending on swimmer’s orientation. (see Fig. F6 of the Supplemental Material [47]). (ii) A close inspection of the results shown in Figs. 4(b) and 4(c) reveals that in agreement with the theory, the variation of net flow speed vs frequency is approximately linear, as was also observed for swimmers in the fluid bulk [see Fig. 3(c)].

Our results indicate that the resulting transverse mean flow is strongly dependent on proximity to the wall, *h*, which naturally raises questions of how one can control this parameter experimentally. Measurements (not reported here) demonstrate that treatment of the chamber bottom surface [such as to modify its charge (hydrophilicity)] can make a major difference, thus controlling the magnitude of such mean flow. In Fig. 4(d), we show results of numerical simulations (using FEM) of the fluid flow generated by the swimmer for different distances *h* [see inset of Fig. 4(d)] from the bottom surface. We used the same configurations as the simulations of flow in the bulk with the addition of a no-slip boundary. As before, we evaluated the steady-state flow profile for 16 angular configurations (0° to 337.5° in 22.5° intervals) of the helix about its axis and averaged over the results to obtain the mean flow field. The representative results for *h* = 1, 1.5, and 2 *μ*m shown in Fig. 4(d) highlight the strong sensitivity of the flow field on *h*. In the simulations we assumed that the swimmer is translating with the experimentally measured propulsion (along its long axis) and drift (sidewise) velocities. We varied *h* between 1 and 2.5 *μ*m at 0.25-*μ*m intervals and subsequently compared the numerical and the experimental results (see Fig. F7 of the Supplemental Material [47]). For frequencies 50 and 40 Hz, *h* = 2 *μ*m provided a good agreement between experimental measurements and numerical simulations of the flow profile, while at lower frequencies between 10 and 30 Hz, *h* = 1.75 *μ*m provided the best agreement between experiments and simulations. As discussed before, this small difference could be due to the variation of the separation distance of the helix from the wall within a single rotation, which in turn is a function of the frequency of rotation.

**Fig. 5:**
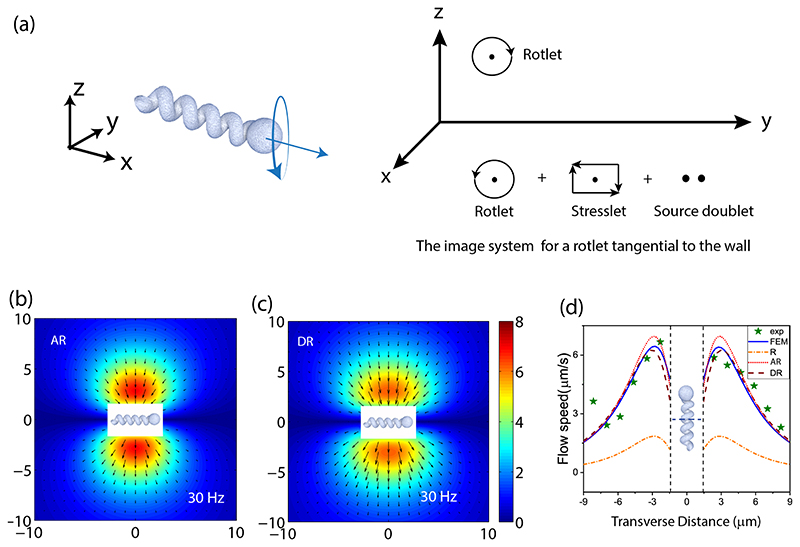
Description of induced flow using multipole representation. (a) Schematic of a point rotlet near a wall and the various image multipoles. (b) Induced flow due to adjusted point rotlet (AR) near a wall (*k* = 3.75) at 30 Hz. (c) Same as (b), except for a line-distributed system of rotlets (DR) parallel to a wall, located along the direction of the stright arrow shown in (a). (d) Comparison of the fluid flow vs the transverse distance in the midplane of the helical structure (see the inset image) for experimental, numerical (FEM), and analytical results due to point rotlet, (*R, k* = 1), AR, *k* = 3.75, and DR.

Next, we model the fluid flow around the rotating microhelix oriented parallel to the wall, following the methodology developed by Blake [49]. Using the method of images, the flow field due to point rotlet acting at the vertical distance *h* above the no-slip boundary at *z* = 0, can be constructed by adding the corresponding images, including a rotlet, a stresslet, and a source doublet [as depicted schematically in Fig. 5(a)]:

(4) $$\begin{array}{l} u_{i}(x)=\frac{T_{j}\epsilon_{ijk}}{8\pi \mu }\left[\frac{r_{k}}{r^{3}}-\frac{R_{k}}{R^{3}}\right]\\ +\frac{T_{j}\epsilon_{kj3}}{8\pi \mu }\left[2h\left(\frac{\delta_{ik}}{R^{3}}-\frac{3R_{i}R_{k}}{R^{5}}\right)+\frac{6R_{i}R_{k}R_{3}}{R^{5}}\right]. \end{array}$$

Here, we assumed that the rotlet is applied at ***x′*** = (*x′*, *y′*, *h*) and the images acting at x′_*_ = (*x*′, *y*′, *h*), so that ***r*** = ***x*** – ***x***′ is the radius vector from the source and ***R*** = ***x*** – ***x***′ is the radius vector from the image. The torque can be expressed as $\frac{T_{j}}{8\pi \eta }=k{\text{Ω}}_{j}a^{3}$, with Ω = 2*πf* and *k* being a fitting parameter to adjust the magnitude of the rotlet (referred as “adjusted point rotlet,” AR, below). Approximating the flow around a microhelix for *h* = 1.75 *μ*m, *a* = 0.6 *μ*m, and *f* = 30 Hz by the adjusted point rotlet [for *k* = 3.75; see Fig. 5(b)], we find a good qualitative resemblance to the experimental observations. However, AR approximation shows some discrepancy in the spatial dependence of the flow on the radial distance [see Fig. 5(d)]. More accurate approximation of the flow can be provided by the continuous (uniform) distribution of rotlets along the helical axis between *x* = –*l* and +*l*. In this case the rotation velocity $\Omega =\text{Ω}\hat{x}\left({\text{or}\;\text{Ω}}_{j}=\text{Ω}\delta_{1j}\right)$ and it follows from Eq. (4) that the fluid velocity components are given by

(5) $$u_{i}(x)=M\int_{-l}^{l}\left(\frac{\epsilon_{i1k}r_{k}}{r^{3}}-\frac{\epsilon_{i1k}R_{k}+2h\delta_{i2}}{R^{3}}-\frac{6R_{i}yz}{R^{5}}\right)dx^{\prime },$$

where ***r*** = (*x*–*x*’, *y*, *z*–*h*), ***R*** = (*x*–*x*’, *y*, *z*+*h*), and $M=\text{Ω}\frac{a^{2}}{2}$ is the constant torque density. Performing the integration and using the approximate boundary conditions at the surface of a cylinder approximating the helix: *u_y_* = –Ω(*z–h*) and *u_z_* = *Ωy* at *y*^2^ + (*z*–*h*)^2^ = *a*^2^ for *–l* ≤ *x* ≤ *l*, we derive explicit closed-form expressions for the flow velocity (see Supplemental Material [47], Sec. S2). The flow field due to distributed rotlets (DR) for *a* = 0.6 *μ*m is shown in Fig. 5(c). While the AR approximation overestimates the maximum flow velocity when compared to the FEM calculations, the model based on DR shows an excellent agreement without any adjustable parameters. It can also be seen in Fig. 5(d) that the point rotlet (*R*, *k* = 1) representation considerably underestimates the magnitude of the flow. In summary, the flow around a rotating helical microswimmer parallel to the wall can be accurately approximated by the line-distributed rotlets and the corresponding images, and this notion is in agreement with previous studies on chemically powered Janus [50] swimmers and bacteria [45].

## IV Conclusions

The presented experimental results, supported by the theory and numerical simulations, demonstrate that driven rotation of the microhelix in a vicinity of a bounding wall yields a strong slowly decaying mean fluid flow in a plane transverse to the helical axis. Such flow is due to the presence of a no-slip boundary, while it is absent in the bulk fluid, where the flow is purely rotational as was also demonstrated in this work. Such mean flow is expected to yield interesting collective dynamics in a swarm of driven microhelices near the surface, as what was reported for the collection of spherical magnetic “microrollers” [51]. The difference is that the microhelices exhibit propulsion (along the axis of the field rotation) owing to their chirality, while the wall contributes to their transverse drift (in plane of the field), while the displacement of spherical rollers (also in plane of the field) is entirely due to the wall proximity. Notice that in the bulk fluid the purely rotational flow around individual helices does not produce any net collective motion [52]. Notice also that while the helical geometry (e.g., pitch) controls the propulsion of the microhelix, it does not have any observable effect on the mean flow. Moreover, in the analytical representation of the flow due to line-distributed rotlets modified by the wall presence (approximating rotation of a cylinder), the helical geometry of the microswimmer is not coming into play, indicating that *propulsion* of the microhelix leaves no (or very small) hydrodynamic signature in the flow field. The same concerns the wall-mediated transverse drift (or sidewise rolling) of the microhelix; its hydrodynamic signature can be safely neglected in describing the mean flow. From theoretical point of view, our results indicate that the prospective contributions of the symmetric force dipole (stresslet) and higher-order singularities (e.g., source doublet) to the flow are weak in comparison with the antisymmetric force-dipole contribution (i.e., rotlets).

To extend these studies to a collection of swimmers, one may also need to consider the magnetic dipole-dipole interactions. These interactions, however, decay as *r*^−3^, faster than the fluidic counterpart (~*r*^−α^, 1 < *α* < 2) and thus are only important at high concentrations [52]. Although we only considered actuation in a purely viscous Newtonian fluid, we believe that a similar analysis should apply to some non-Newtonian fluids, e.g., shear-thinning (-thickening) fluids. It will be interesting to extend the study to viscoelastic [53,54] media and porous media, which are particularly relevant to biomedical applications, e.g., propulsion inside a cell [7,9] or tissue [35].

In conclusion, we have presented a combined experimental and numerical investigation of the flow around a magnetic microhelix rotating in a Newtonian fluid, both in the bulk fluid and near a bounding wall. We showed that flow could be closely approximated by a system of line-distributed rotlets that gets modified close to a wall through appropriate contributions from image multipoles. The analytical representation of the flow around the externally driven microhelix primarily depends on several factors: its geometry (length, *l*, and width, 2*a*), orientation with respect to and distance from the bounding surface (*h*), and the rotation rate (Ω), all of which can be estimated or measured experimentally. The study can help to construct a microscopic theoretical framework of collective dynamics of swarm of driven microhelices near boundaries, which is of relevance to diverse areas ranging from practical applications of targeted drug delivery to fundamental aspects of active matter.

## Supplementary Material

SI

## References

[1] Kagan D, Benchimol MJ, Claussen JC, Chuluun-Erdene E, Esener S, Wang J (2012). Acoustic droplet vaporization and propulsion of perfluorocarbon-loaded microbullets for targeted tissue penetration and deformation. Angew Chem, Int Ed.

[2] Nelson BJ, Kaliakatsos IK, Abbott JJ (2010). Microrobots for minimally invasive medicine. Annu Rev Biomed Eng.

[3] Patra D, Sengupta S, Duan W, Zhang H, Pavlick R, Sen A (2013). Intelligent, self-powered, drug delivery systems. Nanoscale.

[4] Kagan D, Laocharoensuk R, Zimmerman M, Clawson C, Balasubramanian S, Kang D, Bishop D, Sattayasamitsathit S, Zhang L, Wang J (2010). Rapid delivery of drug carriers propelled and navigated by catalytic nanoshuttles. Small.

[5] Gao W, Kagan D, Pak OS, Clawson C, Campuzano S, Chuluun-Erdene E, Shipton E, Fullerton EE, Zhang L, Lauga E, Wang J (2012). Cargo-towing fuel-free magnetic nanoswimmers for targeted drug delivery. Small.

[6] Balasubramanian S, Kagan D, Jack Hu CM, Campuzano S, Lobo-Castañon MJ, Lim N, Kang DY, Zimmerman M, Zhang L, Wang J (2011). Micromachine-enabled capture and isolation of cancer cells in complex media. Angew Chem, Int Ed.

[7] Pal M, Somalwar N, Singh A, Bhat R, Eswarappa SM, Saini DK, Ghosh A (2018). Maneuverability of magnetic nanomotors inside living cells. Adv Mater.

[8] Venugopalan PL, de Ávila BE-F, Pal M, Ghosh A, Wang J (2020). Fantastic voyage of nanomotors into the cell. ACS Nano.

[9] Pal M, Dasgupta D, Somalwar N, VR R, Tiwari M, Teja D, Narayana SM, Katke A, RS J, Bhat R, Saini DK (2020). Helical nanobots as mechanical probes of intra-and extracellular environments. J Phys: Condens Matter.

[10] Sanchez S, Solovev AA, Schulze S, Schmidt OG (2010). Controlled manipulation of multiple cells using catalytic microbots. Chem Commun.

[11] Snezhko A, Aranson IS (2011). Magnetic manipulation of selfassembled colloidal asters. Nat Mater.

[12] Yu J, Wang B, Du X, Wang Q, Zhang L (2018). Ultra-extensible ribbon-like magnetic microswarm. Nat Commun.

[13] Wang B, Chan KF, Yu J, Wang Q, Yang L, Chiu PWY, Zhang L (2018). Reconfigurable swarms of ferromagnetic colloids for enhanced local hyperthermia. Adv Funct Mater.

[14] Mushtaq F, Asani A, Hoop M, Chen X-Z, Ahmed D, Nelson BJ, Pané S (2016). Highly efficient coaxial TiO2-PtPd tubular nanomachines for photocatalytic water purification with multiple locomotion strategies. Adv Funct Mater.

[15] Gao W, Wang J (2014). The environmental impact of mi-cro/nanomachines: A review. ACS Nano.

[16] Ghosh AA, Dasgupta D, Pal M, Morozov KI, Leshansky AM, Ghosh AA (2018). Helical nanomachines as mobile viscometers. Adv Funct Mater.

[17] Fischer P, Ghosh A (2011). Magnetically actuated propulsion at low Reynolds numbers: Towards nanoscale control. Nanoscale.

[18] Ghosh A, Fischer P (2009). Controlled propulsion of artificial magnetic nanostructured propellers. Nano Lett.

[19] Sanchez S, Soler L, Katuri J (2015). Chemically powered micro-and nanomotors. Angew Chem, Int Ed.

[20] Fournier-Bidoz S, Arsenault AC, Manners I, ozin GA (2005). Synthetic self-propelled nanorotors. Chem Commun.

[21] Howse JR, Jones RAL, Ryan AJ, Gough T, Vafabakhsh R, Golestanian R (2007). Self-Motile Colloidal Particles: From Directed Propulsion to Random Walk. Phys Rev Lett.

[22] Gibbs JG, Zhao YP (2009). Autonomously motile catalytic nanomotors by bubble propulsion. Appl Phys Lett.

[23] Dreyfus R, Baudry J, Roper ML, Fermigier M, Stone HA, Bibette J (2005). Microscopic artificial swimmers. Nature (London).

[24] Tierno P, Golestanian R, Pagonabarraga I, Sagués F (2008). Controlled Swimming in Confined Fluids of Magnetically Actuated Colloidal Rotors. Phys Rev Lett.

[25] Luo T, Fan L, Zhu R, Sun D (2019). Microfluidic singlecell manipulation and analysis: Methods and applications. Micromachines.

[26] Cavagna A, Giardina I (2014). Bird flocks as condensed matter. Annu Rev Condens Matter Phys.

[27] Hemelrijk CK, Reid DAP, Hildenbrandt H, Padding JT (2015). The increased efficiency of fish swimming in a school. Fish.

[28] Sambhy V, MacBride MM, Peterson BR, Sen A (2006). Silver bromide nanoparticle/polymer composites: Dual action tunable antimicrobial materials. J Am Chem Soc.

[29] Illien P, Golestanian R, Sen A (2017). Fuelled motion: Phoretic motility and collective behaviour of active colloids. Chem Soc Rev.

[30] Zhang L, Abbott JJ, Dong L, Kratochvil BE, Bell D, Nelson BJ (2009). Artificial bacterial Flagella: Fabrication and magnetic control. Appl Phys Lett.

[31] Morozov KI, Leshansky AM (2014). The chiral magnetic nanomotors. Nanoscale.

[32] Ghosh A, Mandal P, Karmakar S, Ghosh AA (2013). Analytical theory and stability analysis of an elongated nanoscale object under external torque. Phys Chem Chem Phys.

[33] Ghosh A, Paria D, Singh HJ, Venugopalan PL, Ghosh A (2012). Dynamical configurations and bistability of helical nanostructures under external torque. Phys Rev E.

[34] Wang B, Kostarelos K, Nelson BJ, Zhang L (2021). Trends in micro-/nanorobotics: Materials development, actuation, localization, and system integration for biomedical applications. Adv Mater.

[35] Dasgupta D, Pally D, Saini DK, Bhat R, Ghosh A (2020). Nanomotors sense local physicochemical heterogeneities in tumor microenvironments. Angew Chem, Int Ed.

[36] Venugopalan PL, Jain S, Shivashankar S, Ghosh A (2018). Single coating of zinc ferrite renders magnetic nanomotors therapeutic and stable against agglomeration. Nanoscale.

[37] Dasgupta D, Peddi S, Saini DK, Ghosh A (2022). Mobile nanobots for prevention of root canal treatment failure. adv healthcare mater.

[38] Kadiri VM, Bussi C, Holle AW, Son K, Kwon H, Schütz G, Gutierrez MG, Fischer P (2020). Biocompatible nanodevices: Biocompatible magnetic micro-and nanodevices: Fabrication of FePt nanopropellers and cell transfection. Adv Mater.

[39] Ghosh S, Ghosh A (2018). Mobile nanotweezers for active colloidal manipulation. Sci Robot.

[40] Mandal P, Ghosh A (2013). Observation of Enhanced Diffusivity in Magnetically Powered Reciprocal Swimmers. Phys Rev Lett.

[41] Mandal P, Patil G, Kakoty H, Ghosh A (2018). Magnetic active matter based on helical propulsion. Acc Chem Res.

[42] Schamel D, Mark AG, Gibbs JG, Miksch C, Morozov KI, Leshansky AM, Fischer P (2014). Nanopropellers and their actuation in complex viscoelastic media. ACS Nano.

[43] Walker D, Käsdorf BT, Jeong HH, Lieleg O, Fischer P (2015). Biomolecules: Enzymatically active biomimetic micropropellers for the penetration of mucin gels. Sci Adv.

[44] Peyer KE, Zhang L, Nelson BJ (2011). Localized non-contact manipulation using artificial bacterial flagella. Appl Phys Lett.

[45] Drescher K, Dunkel J, Cisneros LH, Ganguly S, Goldstein RE (2011). Fluid dynamics and noise in bacterial cell-cell and cell-surface scattering. Proc Natl Acad Sci USA.

[46] Venugopalan PL, Sai R, Chandorkar Y, Basu B, Shivashankar S, Ghosh A (2014). Conformal cytocompatible ferrite coatings facilitate the realization of a nanovoyager in human blood. Nano Lett.

[48] Chwang AT, Wu TYT (1974). Hydromechanics of low-Reynolds-number flow. Part 1. Rotation of axisymmetric prolate bodies. J Fluid Mech.

[49] Blake JR (1971). A note on the image system for a Stokeslet in a no-slip boundary. Math Proc Cambridge Philos Soc.

[50] Campbell AI, Ebbens SJ, Illien P, Golestanian R (2019). Experimental observation of flow fields around active Janus spheres. Nat Commun.

[51] Driscoll M, Delmotte B, Youssef M, Sacanna S, Donev A, Chaikin P (2017). Unstable fronts and motile structures formed by microrollers. Nat Phys.

[52] Morozov KI, Leshansky AM (2020). Towards focusing of a swarm of magnetic micro/nanomotors. Phys Chem Chem Phys.

[53] Ghosh A, Ghosh A (2021). Mapping viscoelastic properties using helical magnetic nanopropellers. Trans Indian Natl Acad Eng.

[54] Nelson BJ, Peyer KE (2014). Micro-and nanorobots swimming in heterogeneous liquids. ACS Nano.

