# Supplementary material for: Fluid flow induced by helical microswimmers in bulk and near walls
Source: Phys Rev Res. Author manuscript; Available in PMC 2023 Jun 4. (PMC7614617; doi:10.1103/PhysRevResearch.4.033069)
Supplement: SI [file EMS176465-supplement-SI.pdf]

Supplemental Material

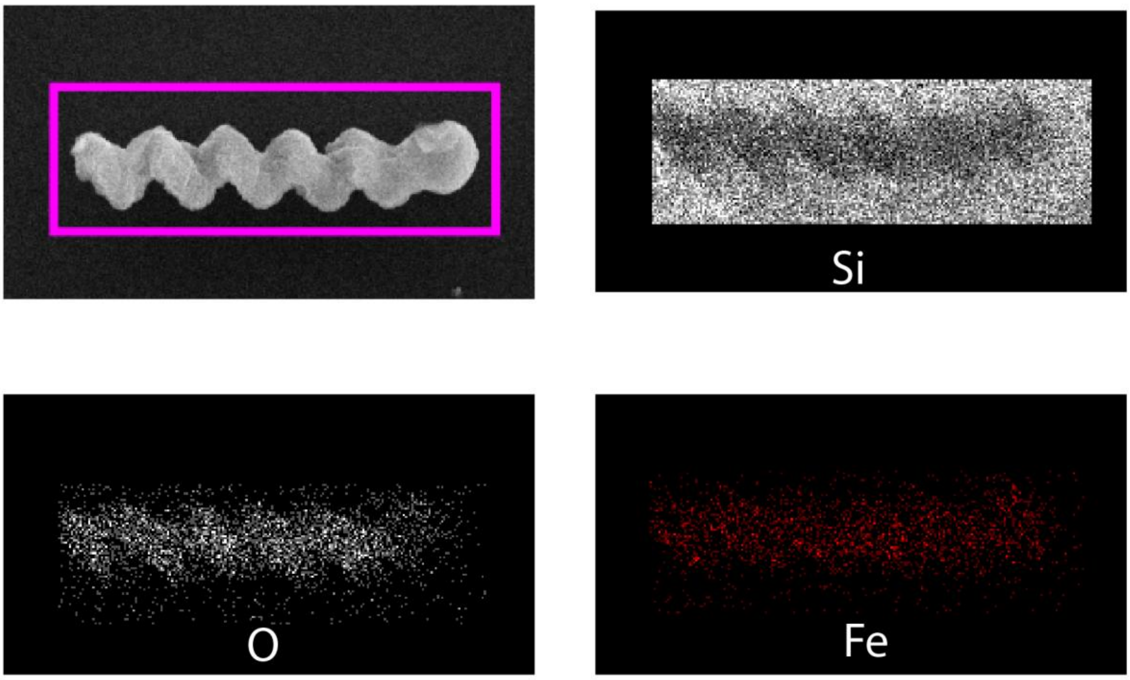

Figure F1: Energy dispersive spectroscopy (EDS) scanning electron micrograph of the microswimmer showing distribution of the magnetic material along the body of the helix.

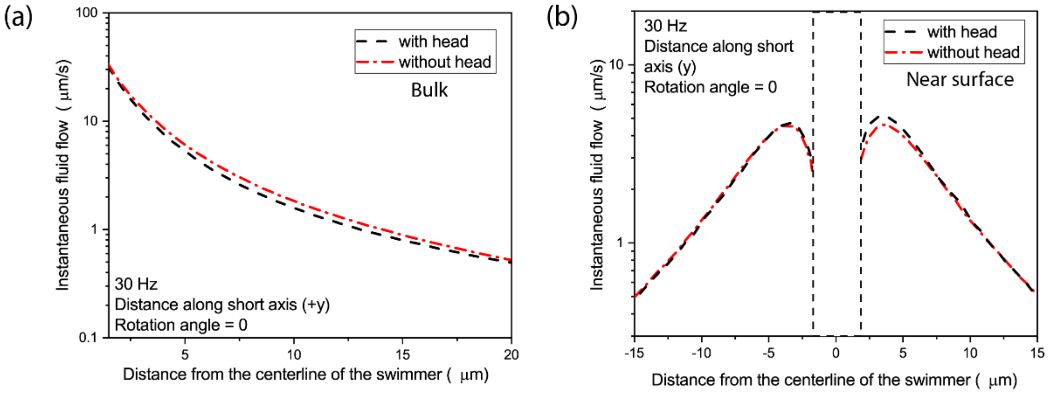

Figure F2: Comparison of the fluid flow simulated using FEM for helical geometry with and without head. Shown are the fluid flows for swimmers rotating at 30 Hz (a) in bulk, an (b) near surface.

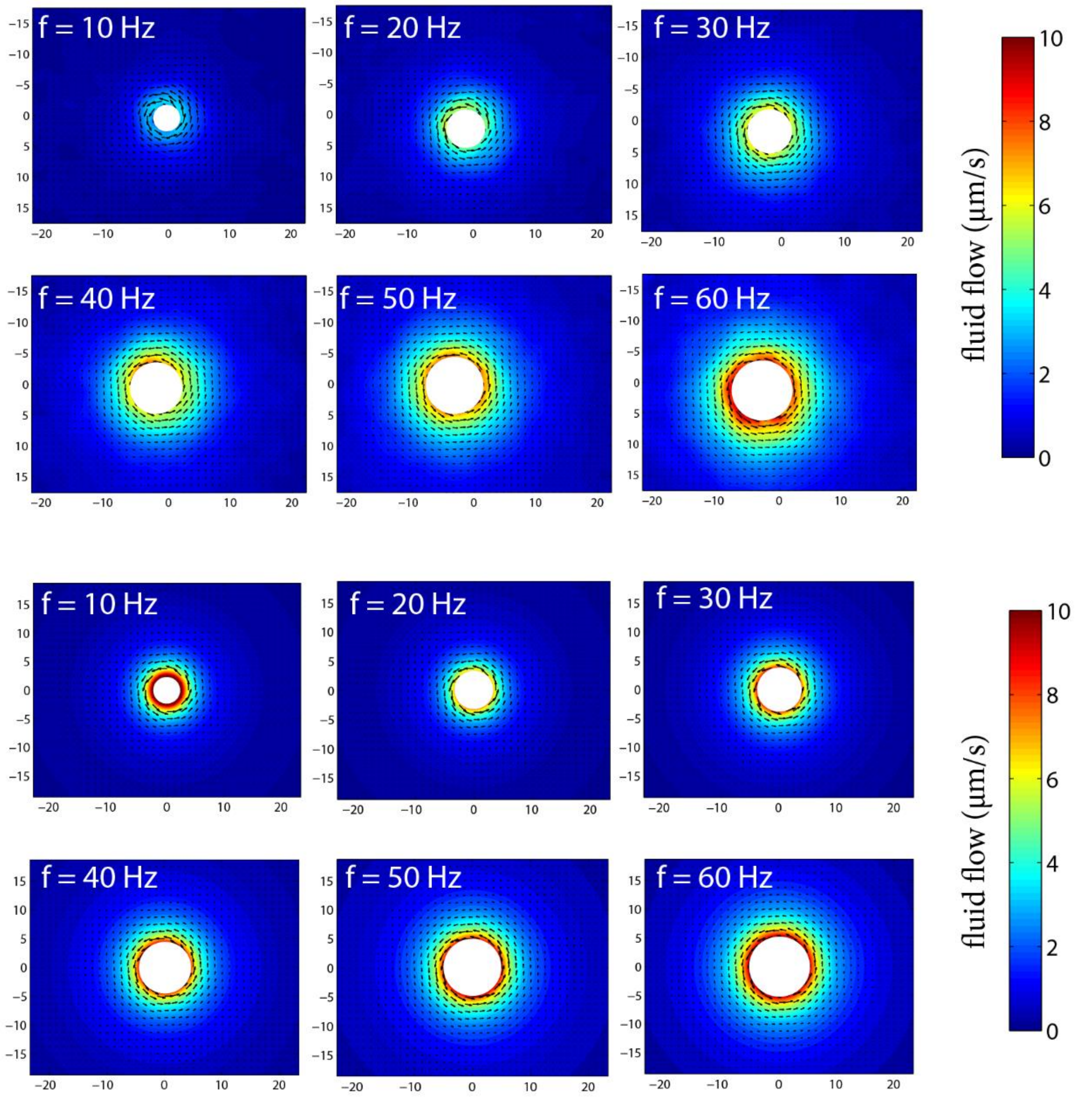

Figure F3: (a) Experimental and (b) simulated fluid flow profiles for microswimmers rotating at different frequencies in the fluid bulk

Section S1: Derivation of fluid flow for distributed system of rotlets in the fluid bulk

Consider a helix of diameter $2a$ rotating around its long z-axis with angular velocity $\mathbf{\Omega} = \Omega\hat{\boldsymbol{z}}$ in fluid bulk. The centerline of the helix to be given by $-l \leq z \leq l,\ y = 0,\ x = 0$. We further assume, that the flow induced by the rotating helix can be closely approximated by the flow around a cylinder of the length $2l$ and radius a, enclosing the helix and turning at the same rate. The boundary condition at the surface of the cylinder at $y^2 + x^2 = a^2$ for $-l \leq z \leq l$ is $u_x = -\Omega y$ and $u_y = \Omega x$. The velocity field is then given by a line distribution of rotlets along the cylinder axis and it can be written as:

$$u_i(\boldsymbol{x}) = \int_{-l}^{l} M_j \, G_{ij} \, dz \,,$$

where the kernel $G_{ij} = \frac{\epsilon_{ijk} r_k}{r^3}$, with $\boldsymbol{r} = (x,\ y,\ z - z')$ and M_j being the torque density. Therefore, assuming uniform torque density, $M_j = \delta_{j3} M,$ we readily obtain:

$$u_x = -M \int_{-l}^{l} \frac{y \, dz'}{\{x^2 + y^2 + (z - z')^2\}} = -M \frac{y}{x^2 + y^2} \left\{ \frac{l - z}{\sqrt{x^2 + y^2 + (l - z)^2}} + \frac{l + z}{\sqrt{x^2 + y^2 + (l + z)^2}} \right\}$$

Similarly, one can readily find:

$$u_y = M \frac{x}{x^2 + y^2} \left\{ \frac{l - z}{\sqrt{x^2 + y^2 + (l - z)^2}} + \frac{l + z}{\sqrt{x^2 + y^2 + (l + z)^2}} \right\},$$

$$u_z = 0 \,.$$

It can be readily seen that far from the ends of the cylinder, $l \pm z \gg a$ the above velocity field satisfies the boundary conditions $u_x \approx -\Omega y$ and $u_y \approx \Omega x$ at the surface $y^2 + x^2 = a^2$ for $-l \ \le \ z \ \le \ l$ for $M = \ \Omega \frac{a^2}{2}$.

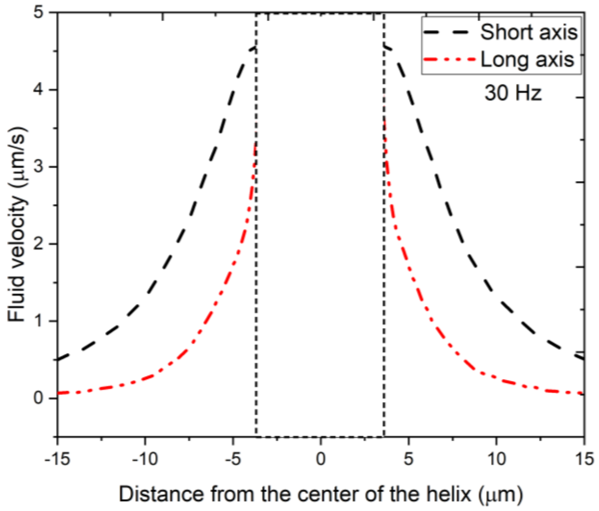

Figure F4: Comparison of the fluid flow along and perpendicular to the helical axis in the numerical simulation. Geometry of the helix is provided in the main text.

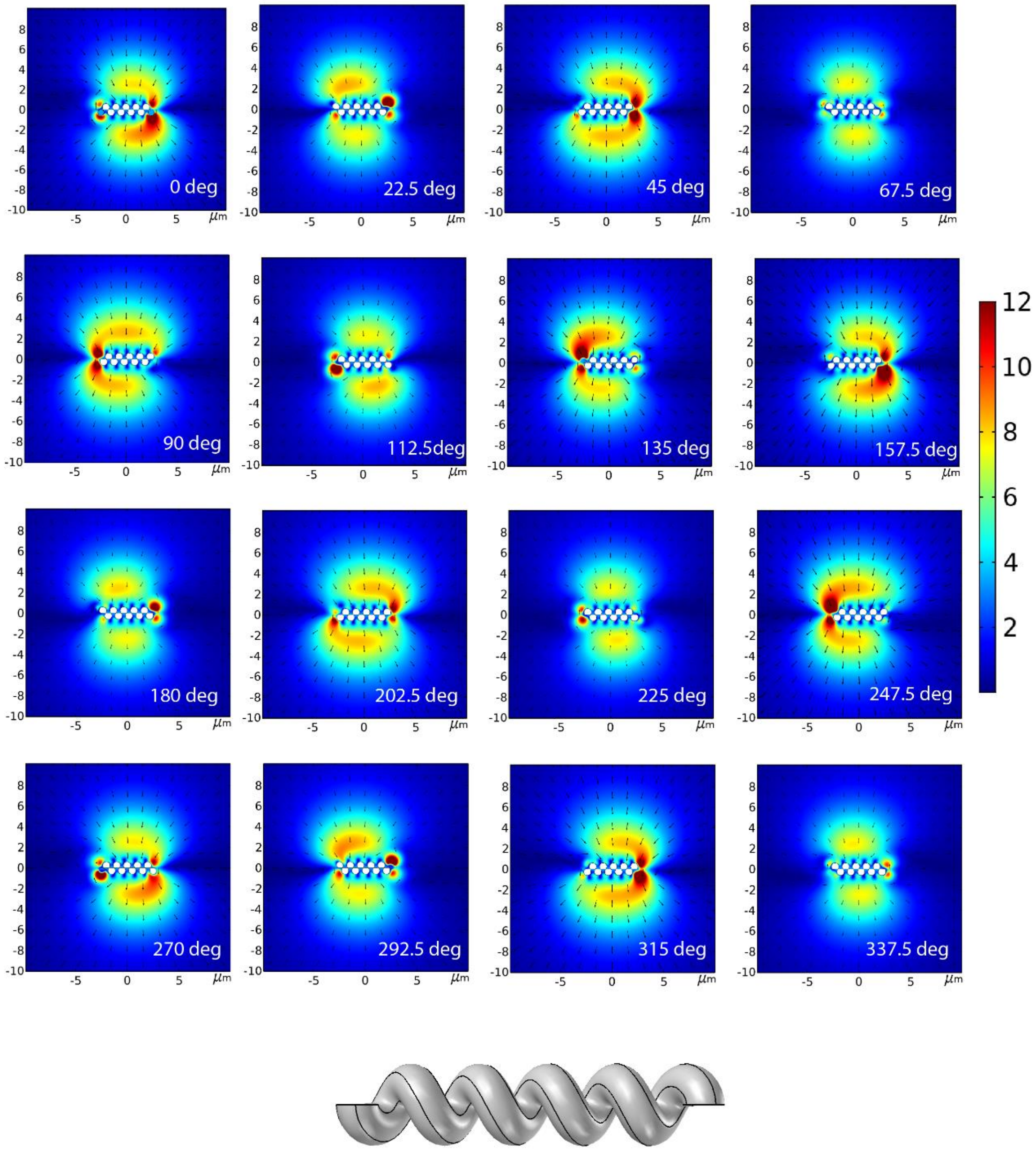

Figure F5: Instantaneous fluid flow profiles (numerical simulation) at different turning angles. The turning angle is shown at the right bottom corner of each plot. The propulsion speed and the sideways drift velocity in the simulation were 2.65 µm/s and 2.18 µm/s, respectively. Distance from the centre of the helix to the wall is 1.5 µm and frequency of rotation 30 Hz.

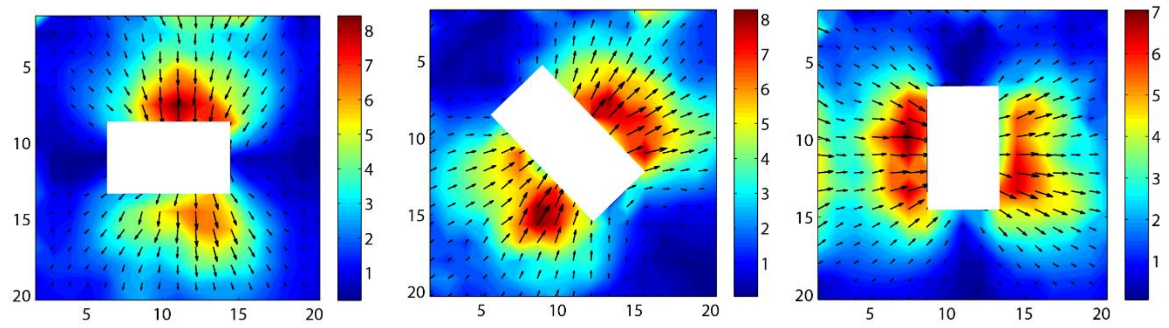

Figure F6: Change in the fore-and-aft asymmetry of net fluid flow due to suggested misalignment of the bottom wall and the imaging plane upon varying orientation of the swimmer.

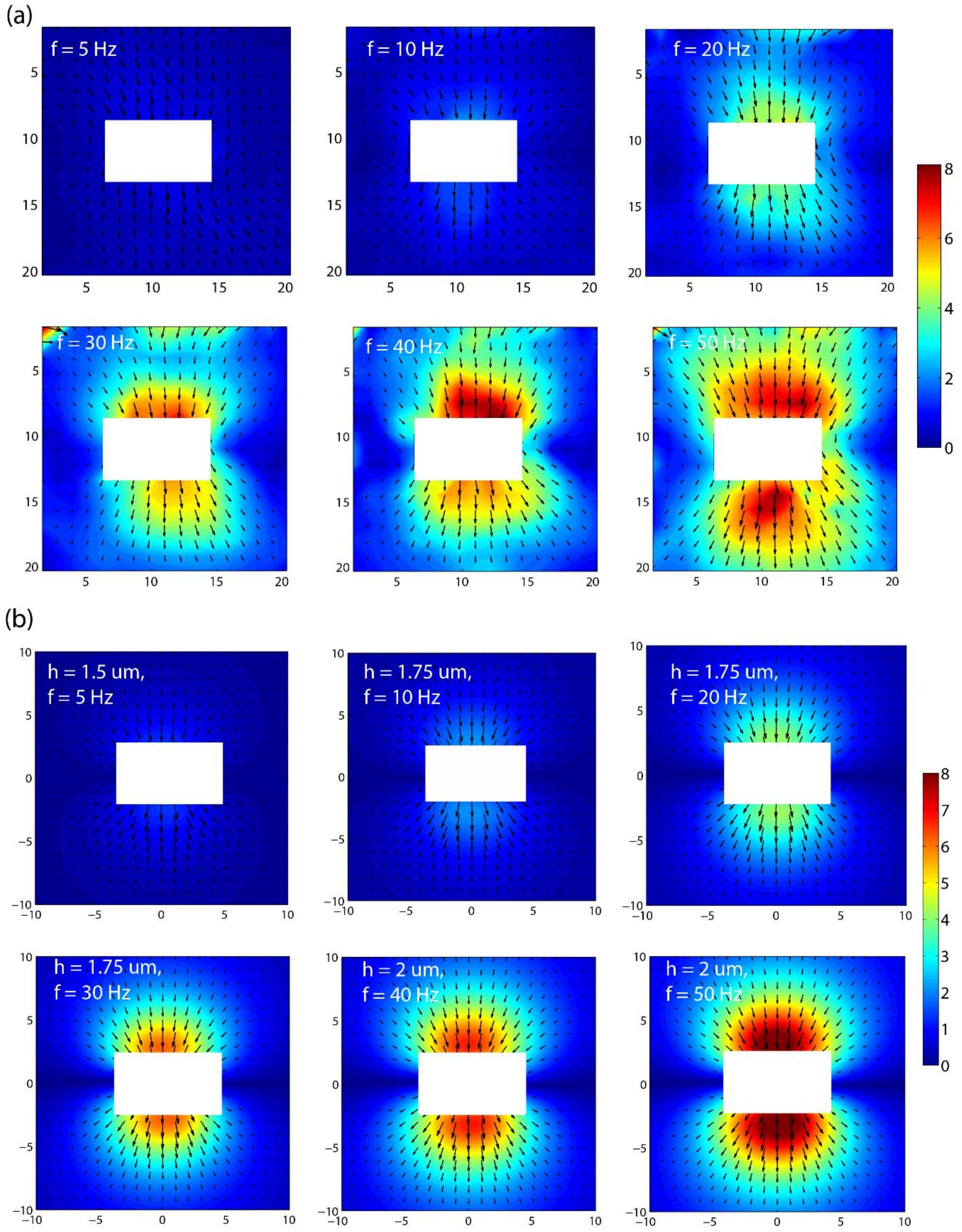

Figure F7: (a) Experimental profiles at different rotation rates. (b) Simulated profiles for different values of h and rotation rates 5 – 50 Hz. The propulsion speed and the sideways drift velocity in the simulation were 2.65 μm/s and 2.18 μm/s, respectively.

Section S2 Derivation of fluid flow due to the system of rotlets line-distributed parallel to the wall

We consider the flow field around a helix rotating around its helical axis which is parallel to the adjacent bounding rigid wall. As done before, we assume that the flow can be approximated by that due to a rotating cylinder enclosing the helix. The cylinder centreline is given by $-l \leq x \leq l,\ y = 0$ and $z = h$ is the vertical distance between the cylinder axis and the wall. The angular velocity is thus $\boldsymbol{\Omega} = \Omega\hat{\boldsymbol{x}}$. The corresponding no-slip boundary conditions at the surface of the cylinder at $y^2 + (z-h)^2 = a^2$ for $-l \leq x \leq l$ are $u_y = -\Omega(z-h)$ and $u_z = \Omega y$. We look for the solution in the form (see Eq.5 in the main text):

$$u_i = M\int_{-l}^{l} \left(\frac{\epsilon_{i1k}r_k}{r^3} - \frac{\epsilon_{i1k}R_k + 2h\delta_{i2}}{R^3} - \frac{6R_i yz}{R^5}\right) dx' ,$$

where $\boldsymbol{r} = (x-x', y, z-h)$, $\boldsymbol{R} = (x-x', y, z+h)$ and M is the constant torque density. It can be readily shown that this solution satisfies $u_i = 0$ at the bounding wall at $z = 0$. Substituting $i = 1$ we obtain the x-component of the flow as:

$$u_x(\boldsymbol{x}) = 6yzM \int_{-l}^{l} \frac{(x-x')dx'}{[(x-x')^2 + y^2 + (z+h)^2]^{5/2}}$$
$$= 2yzM\left[\frac{1}{\{(x+l)^2 + y^2 + (z+h)^2\}^{3/2}} - \frac{1}{\{(x-l)^2 + y^2 + (z+h)^2\}^{3/2}}\right].$$

Notice that $u_x(\boldsymbol{x})$ vanishes for an infinite cylinder as $l \to \infty$, while for finite cylinder the longitudinal flow is entirely due to images. The y-component of the fluid velocity is:

$$u_y(\boldsymbol{x}) = M\int_{-l}^{l} \left(\frac{h-z}{r^3} - \frac{h-z}{R^3} - \frac{6y^2z}{R^5}\right) dx' .$$

Making use of $\int \frac{dx}{(x^2+y^2)^{3/2}} = \frac{x}{y^2\sqrt{x^2+y^2}}$, we find that

$$\int_{-l}^{l} \frac{dx'}{r^3} = \frac{dx'}{[x'^2 + y^2 + (z-h)^2]^{3/2}}$$
$$= \frac{l-x}{[y^2 + (z-h)^2][(l-x)^2 + y^2 + (z-h)^2]^{1/2}}$$
$$+ \frac{l+x}{[y^2 + (z-h)^2][(l+x)^2 + y^2 + (z-h)^2]^{1/2}}.$$

Analogously we have:

$$\int_{-l}^{l} \frac{dx'}{R^3} = \frac{l-x}{[y^2 + (z+h)^2][(l-x)^2 + y^2 + (z+h)^2]^{1/2}}$$
$$+ \frac{l+x}{[y^2 + (z+h)^2][(l+x)^2 + y^2 + (z+h)^2]^{1/2}}.$$

Finally, making use of the integral

$$\int \frac{dx}{(x^2+y^2)^{5/2}} = \frac{2x^3+3xy^2}{3y^4(x^2+y^2)^{3/2}},$$

we the last term gives:

$$\int_{-l}^{l} \frac{dx'}{R^5} = \frac{2(l-x)^3+3(l-x)[y^2+(z+h)^2]}{3[y^2+(z+h)^2]^2[(l-x)^2+y^2+(z+h)^2]^{3/2}} + \frac{2(l+x)^3+3(l+x)[y^2+(z+h)^2]}{3[y^2+(z+h)^2]^2[(l+x)^2+y^2+(z+h)^2]^{3/2}}.$$

Collecting all three integrals we obtain:

$$\begin{aligned} u_y = M(h-z)\Bigg[&\frac{l-x}{\{y^2+(z-h)^2\}\{(l-x)^2+y^2+(z-h)^2\}^{1/2}} \\ &+ \frac{l+x}{\{y^2+(z-h)^2\}\{(l+x)^2+y^2+(z-h)^2\}^{1/2}}\Bigg] \\ &- M(h-z)\Bigg[\frac{l-x}{\{y^2+(z+h)^2\}\{(l-x)^2+y^2+(z+h)^2\}^{1/2}} \\ &+ \frac{l+x}{\{y^2+(z+h)^2\}\{(l+x)^2+y^2+(z+h)^2\}^{1/2}}\Bigg] \\ &- 6My^2z\Bigg[\frac{2(l-x)^3+3(l-x)(y^2+(z+h)^2)}{3\{y^2+(z+h)^2\}^2\{(l-x)^2+y^2+(z+h)^2\}^{3/2}} \\ &+ \frac{2(l+x)^3+3(l+x)(y^2+(z+h)^2)}{3\{y^2+(z+h)^2\}^2\{(l+x)^2+y^2+(z+h)^2\}^{3/2}}\Bigg]. \end{aligned}$$

Assuming that $h \gg a$, at the surface of the cylinder far from its ends, $l \pm x \gg a$, we have

$$u_y(\boldsymbol{x}) \approx \frac{2M(h-z)}{a^2},$$

which gives $M \approx \Omega\frac{a^2}{2}$ from the boundary condition, resulting in the same torque density as in the case of a cylinder rotating in the bilk fluid.

Finally, we consider the vertical component of the flow perpendicular to the wall:

$$u_z(\boldsymbol{x}) = M\int_{-l}^{l}\left(\frac{y}{r^3} - \frac{y}{R^3} - \frac{6yz(z+h)}{R^5}\right)dx'.$$

Performing integration similar to what was done before, yields the following result:

$$\begin{aligned} u_z(\boldsymbol{x}) = yM\Bigg[&\frac{l-x}{\{y^2+(z-h)^2\}\{(l-x)^2+y^2+(z-h)^2\}^{1/2}} \\ &+ \frac{l+x}{\{y^2+(z-h)^2\}\{(l+x)^2+y^2+(z-h)^2\}^{1/2}}\Bigg] \\ -yM\Bigg[&\frac{l-x}{\{y^2+(z+h)^2\}\{(l-x)^2+y^2+(z+h)^2\}^{1/2}} \\ &+ \frac{l+x}{\{y^2+(z+h)^2\}\{(l+x)^2+y^2+(z+h)^2\}^{1/2}}\Bigg] \end{aligned}$$

$$
-6Myz(z+h)\left[\frac{2(l-x)^3+3(l-x)(y^2+(z+h)^2)}{3\{y^2+(z+h)^2\}^2\{(l-x)^2+y^2+(z+h)^2\}^{3/2}}\right.
$$

$$
\left.+\frac{2(l+x)^3+3(l+x)(y^2+(z+h)^2)}{3\{y^2+(z+h)^2\}^2\{(l+x)^2+y^2+(z+h)^2\}^{3/2}}\right].
$$

At the surface of the cylinder $y^2+(z-h)^2=a^2$, far from its ends, $l\pm x\gg a$, we have in the leading approximation

$$
u_z(\boldsymbol{x})\approx\frac{2yM}{a^2},
$$

which gives for $M=\Omega\frac{a^2}{2}$ the correct boundary condition $u_z\approx\Omega y$.
